# 

**Published:** 1889-07-20

**Authors:** 


SATURDAY, JULY 20th, 1889.

**English Hotels and their Critics** - 239
**A Physiological Sunday.** By the Rev. T. G. HEADLEY - 240
**The Doctor's Pulpit.**—In Nature's School - 241
**Hospitals without Medical Schools.**—Unconsidered Trifles - 242
**The Student's Column.**—Medical Jurisprudence for India—University of London M.B. Pass Examination - 243
**The Hospital Workshop.**—No. VI. At the Royal Westminster Ophthalmic Hospital - 244
**Editor's Letter-Box.**—English Hotels and their Critics - 245
**Notes and News** - 246
**Everybody's Page** - 248
**Annotations.**—Barmaids in Council—A New Brain Wanted—Men or Women - 249
**Within the Wards.**—Leprosy in England - 250
**Her Heart's Mistake.** By E. D. CUMMING. - 251
**Turning Over New Leaves.**—Mr. Froud as a Novelist - 253
**Prize Day at the London** - 253
**Amusements and Relaxation** - 254
**Scraps and Gleanings** - 254

EXTRA SUPPLEMENT—THE NURSING MIRROR.

**En Passant.**—Nurses' Friends—Registration—Medical Women for India—Moral Atmosphere—Meeting at the Mansion House—Prize-giving at the London—Nurses at a Fete lix
**The Proposed Registration of Nurses.**—Memorial of Nurse-Training School Authorities - lx
**The British Nurses' Association** - lx
**Physiology for Nurses.**—No. 2. The Skin - lxi
**The National Pension Fund for Nurses** - lxi
**Everybody's Opinion.**—The late Accident at the Rotunda lxii
**Appointments—Vacancies—Notes and Queries** - lxii

